# Producer vs. parental cell – metabolic changes and burden upon α_1_-antitrypsin production in AGE1.HN^®^

**DOI:** 10.1186/1753-6561-5-S8-P85

**Published:** 2011-11-22

**Authors:** Jens Niklas, Christian Priesnitz, Volker Sandig, Thomas Rose, Elmar Heinzle

**Affiliations:** 1 Biochemical Engineering Institute, Saarland University, 66123 Saarbrücken, Germany; 2 ProBioGen AG, 13086 Berlin, Germany

## Background

The human designer cell line AGE1.HN^®^ represents a promising production system for biopharmaceuticals, particularly for those needing human-type post-translational modifications [1,2]. For further rational improvement of the cell line and the cultivation process a detailed understanding of the metabolism and metabolic changes during glycoprotein production is desirable. Metabolism and metabolic burden upon production of α_1_-antitrypsin (A1AT) were analyzed by comparing parental cells and a derived clone, AGE1.HN.AAT. The questions addressed were (i), which changes occur in cell growth and metabolism upon A1AT production and (ii), what are specific cellular properties distinguishing the producer clone AGE1.HN.AAT from the parental cell population.

## Materials and methods

### Cultivation and analysis

Cultivations of the human neuronal cell line AGE1.HN® (ProBioGen AG, Berlin, Germany) were carried out in shake flasks (Corning, NY, USA). Extracellular metabolites were quantified using different HPLC methods [3,4]. A1AT was quantified using an activity assay. Identification of extracellular proteins was done using MALDI-ToF/ToF MS (Applied Biosystems). Biomass components were quantified using classical biochemical methods.

### Model for A1AT production

Model for A1AT production was set up using the Kyoto Encyclopedia of Genes and Genomes (http://www.kegg.com) pathway database for Homo sapiens.

### Metabolic flux analysis

Fluxes were estimated using standard methods [1,5] by incorporating extracellular and anabolic rates using Matlab R2007b (The Mathworks, Natick, MA, USA).

## Results

Cell growth was similar in the first 5 days. After 5 days AGE1.HN.AAT cells were growing less than the parental cell line and the viability dropped faster. After 2 days dry weight was slightly higher in the parental cell line. A1AT production was low in the first 2 days and then increasing up to a final concentration of ~0.4 g/l in the end of the cultivation. The total cell mass was increasing similarly in the first 4 days. After 4 days AGE1.HN.AAT cell mass remained constant whereas the extracellular protein concentration was increasing. This was different in the AGE1.HN parental cells where the cell mass was still increasing after 4 days and the increase in total extracellular protein mass was less. Intracellular protein concentration was decreasing in AGE1.HN.AAT after 4 days. Specific cellular proteins might be degraded upon nutrient deprivation to maintain the production of the recombinant glycoprotein A1AT. This was clearly different in the parental cell line. Fractions of the biomass constituents RNA, lipid and phosphatidylcholine were increased in AGE1.HN.AAT. The total mass of the analyzed components in the end of the cultivation was similar in the cultivations of both cell lines being around 2.5 g/l. The final A1AT concentration which was 0.4 g/l was ~30 % of the total protein in the culture.

Similar time courses for most extracellular metabolites were observed. Glycine and glutamate production was higher in AGE1.HN.AAT whereas uptake of arginine and aspartate as well as alanine production were lower. Glutamine was consumed in the cultivations of both cell lines at ~4 days which resulted also in reduced growth. Especially the increase in glycine and glutamate production points to differences in C1 metabolism and cellular nucleotide demand.

**Figure 1:**
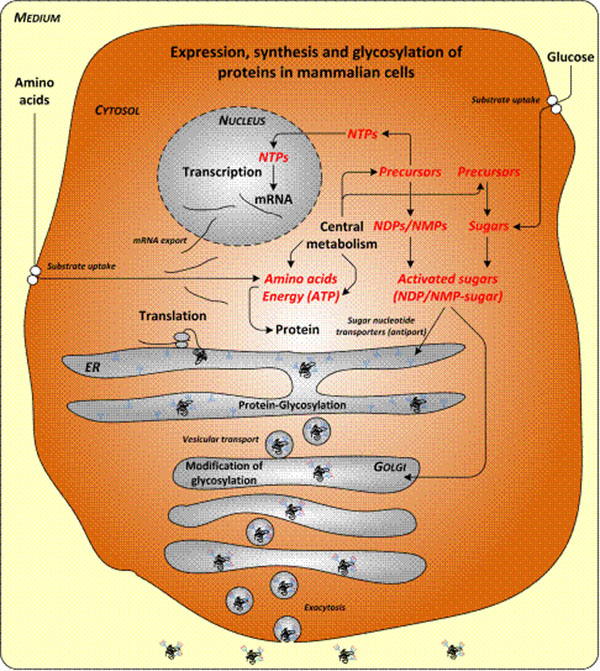
Schematic presentation of the glycoprotein production process in mammalian cells.

The differences that can be seen in the metabolism upon production of the glycoprotein were explained by using an appropriate model of the anabolic demand needed to produce active A1AT. The whole cellular production process of a glycoprotein includes its expression (transcription), synthesis of the amino acid chain (translation), posttranslational modification in endoplasmic reticulum (ER) and Golgi apparatus (Golgi) and secretion of the mature protein (Figure 1). Metabolite demand for A1AT production was finally simulated. These results indicate that one could expect an increase in the production of glutamate and glycine with increasing intracellular nucleotide demand which was observed for AGE1.HN.AAT; this is caused by differences in cellular composition of the producer, partly originating from recombinant A1AT production.

The differences that were found in the biomass composition between both cell lines were included in a metabolic network model and intracellular metabolic fluxes were calculated for both cell lines. Fluxes in central energy metabolism (glyolysis, TCA cycle) were similar. Differences were observed in C1 and glutamate metabolism including changes in activities of several transaminases. The observed changes in metabolic flux reflect the anabolic differences between producer and parental cells.

## Conclusions

This improved understanding of the metabolism and cellular changes during glycoprotein production supports the identification of targets for further improvement of (i) cell line, e.g., by genetic modifications and (ii) cultivation process, e.g., by improved feeding strategies. The presented data indicate that nucleotide and lipid metabolism might be interesting targets for further engineering of the AGE1.HN^®^ cell line.
